# Reducing Cadmium Accumulation in Plants: Structure–Function Relations and Tissue-Specific Operation of Transporters in the Spotlight

**DOI:** 10.3390/plants9020223

**Published:** 2020-02-09

**Authors:** Xin Huang, Songpo Duan, Qi Wu, Min Yu, Sergey Shabala

**Affiliations:** 1 International Research Center for Environmental Membrane Biology, Foshan University, Foshan 528000, China; Xin.Huang@utas.edu.au (X.H.); songpo.duan@hotmail.com (S.D.); qi.wu@fosu.edu.cn (Q.W.); yumin@fosu.edu.cn (M.Y.); 2 Tasmanian Institute of Agriculture, University of Tasmania, Hobart TAS 7001, Australia

**Keywords:** cadmium toxicity, membrane transport, tissue tolerance, NRAMP, IRT, MTP, non-selective cation channel

## Abstract

Cadmium (Cd) is present in many soils and, when entering the food chain, represents a major health threat to humans. Reducing Cd accumulation in plants is complicated by the fact that most known Cd transporters also operate in the transport of essential nutrients such as Zn, Fe, Mn, or Cu. This work summarizes the current knowledge of mechanisms mediating Cd uptake, radial transport, and translocation within the plant. It is concluded that real progress in the field may be only achieved if the transport of Cd and the above beneficial micronutrients is uncoupled, and we discuss the possible ways of achieving this goal. Accordingly, we suggest that the major focus of research in the field should be on the structure–function relations of various transporter isoforms and the functional assessment of their tissue-specific operation. Of specific importance are two tissues. The first one is a xylem parenchyma in plant roots; a major “controller” of Cd loading into the xylem and its transport to the shoot. The second one is a phloem tissue that operates in the last step of a metal transport. Another promising and currently underexplored avenue is to understand the role of non-selective cation channels in Cd uptake and reveal mechanisms of their regulation.

## 1. Cd Toxicity as an Issue

### 1.1. Cadmium in Soils

Cadmium (Cd), a non-essential metal element for people and plants, is widely present in soils in Europe, China, Japan, and America [1]. The origin of cadmium can be divided into two types; one is from natural sources, the other one is of anthropogenic origin [2]. While cadmium is also present in the air and water, most of it eventually goes to the soil. Mineral oil and heavy metals are the main pollutants causing soil contamination, accounting for about 60%. In nature, Cd is released into the environment through rivers and the atmosphere, mainly from weathering of soil parent material and volcanic activities, at an estimated rate of about 820 metric tons per year [3,4]. In contrast, anthropogenic Cd emissions are more serious, accounting for 8000 to 10,000 mt per year [5]. The major sources of anthropogenic Cd are from cadmium-related manufacturing, application of chemical fertilizers, contaminated sewage sludge and waste water, sewage effluents, and agricultural run-offs [6]. Cadmium migrates into the surrounding soil and atmosphere through solid waste combustion. Compared with urban soils, the content of cadmium in agricultural soil is relatively low [7]. However, due to the intensive use of phosphate fertilizer or leakage of factory sewage, Cd accumulation in agricultural soils is becoming a major issue. Cadmium is of a particular concern for plants, because it accumulates in leaves in high quantities, which can be 10–500 times higher than in plants grown in non-polluted environments, and may then enter the food chain by being eaten by animals or humans [8]. The ability of plants to absorb cadmium mainly depends on the concentration of soil Cd and plant ability of accumulation and translocation, and is also affected by pH, temperature, and redox potential, as well as by concentrations of other elements and soil organic matter [9]. For example, excessive use of nitrogen and phosphate fertilizer in many areas of China led to increased soil acidification, while acidic soil accelerates the absorption of cadmium by plants [10]. Drought stress may also increase the rate of cadmium uptake 2–3-fold, compared with non-stressed conditions [11,12]. As described in Section 3.1, Cd uptake by plant roots shares the same pathway with zinc (Zn). Zn is an essential micronutrient and usually accompanies Cd with a ratio of 100:1 in most natural systems and anthropogenic environments [13,14,15]. Due to their similar geochemistry, Cd and Zn interactions are often of a synergistic nature. However, Cd toxicity is almost 10 times higher than that of Zn [16,17], and Cd is not required for plant growth and development, while Zn is considered essential for many metabolic functions.

### 1.2. Cadmium Toxicity: Health Implications

Most studies have shown that excessive cadmium accumulation leads to kidney dysfunction and lung damage. Cadmium is also a carcinogen that can cause kidney cancer and breast canceranemia. Other negative health effects include heart failure, hypertension, cerebral infarction, proteinuria, eye cataract formation, osteoporosis, emphysema, and renal insufficiency [18,19]. For example, the average weekly cadmium accumulation in Japan during the 1980s to 2000s was up to 4 mg Cd per kilogram of body weight [20]; this has resulted in an outbreak of the Itai-Itai disease caused by Cd contamination in rice in the river basin of Toyama [6,21]. In a study on rats, a high level of lipid peroxidation was observed under the continued oral application of 3.5 mg∙kg^−1^ of Cd, resulting in a high MDA level in liver, kidney, and serum [22]. Acute Cd treatments at higher doses resulted in a massive and rapid hepatic necrosis [23]. Cadmium accumulates in the human body mainly through the food chain, and most food comes from edible parts or seeds of crops [24]. An estimated 98% of cadmium intake comes from terrestrial foods, only 1% from aquatic foods, and 1% from cadmium in a drinking water [25]. Plant foods are generally considered to be the main source of Cd exposure in the population, and grains account for a large proportion of the total dietary intake. Wheat and rice are the major food crops for the global population [26,27]. Both species can easily absorb cadmium from the soil and accumulate it in grains [28,29]. In China, the average intake of cadmium for the general population more than doubled between 1990 and 2015 [30,31]. Of a specific concern is also Cd accumulation in leafy vegetables. Lettuce (*Lactuca sativa* L.) and endive (*Cichorium endivia* L.) are two important salad leafy vegetables; both may accumulate significant amounts of Cd in edible parts that can then enter the food chain. In these species, some studies have reported Cd contents of up to 9 mg∙kg^−1^ in their leaves, fourfold higher than that in roots and 20–30-fold higher than the actual Cd content in the soil (Table 1) [32,33]. Thus, preventing excessive accumulation of Cd in grains and leafy vegetables is critical for population health [34].

### 1.3. Cadmium Impact on Plant Growth and Performance

**Figure 1:**
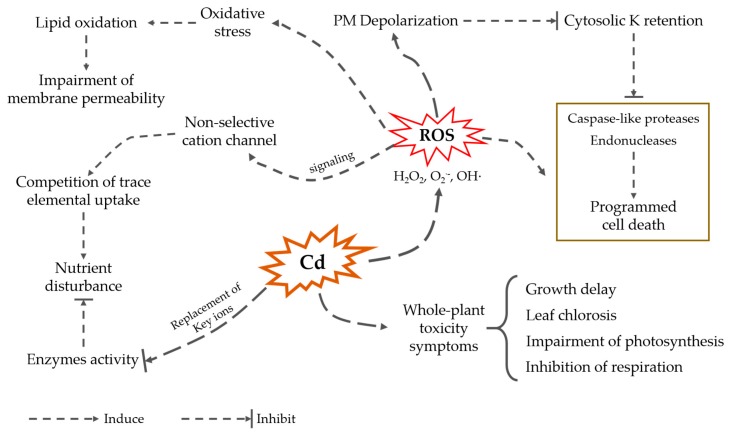
Cadmium effect on plant metabolism and growth.

Cadmium causes a wide range of deleterious effects on plants, affecting plant metabolism and causing oxidative stress, nutrient uptake disturbance, and even plant death (Figure 1; [35]). At the whole-plant level, symptoms of Cd toxicity include growth delay, leaf chlorosis, and inhibition of photosynthesis and respiration [36]. Cd can impair plant development and growth by interfering with biochemical and physiologically related signaling pathways, such as affecting photosynthesis by interfering with photosynthetic electron transport, resulting in decreased chlorophyll content and stomatal conductance [37]. Moreover, the inhibition of root Fe(III) reductase induced by Cd led to Fe(II) deficiency, and it seriously affected photosynthesis [38]. Cadmium also changes membrane permeability, leading to a decrease in water content and affecting water balance [39]. 

Cadmium is a non-essential element; thus, plants have not developed a specialized uptake system for its absorption. Instead, Cd enters plants and is transported across various membranes by other metal transporters [40,41,42,43] or non-selective cation channels [44,45]. This results in a competition with acquisition of some essential metals. In addition, cadmium can also replace ions on the active sites of some enzymes, thus disrupting their activity and absorption of essential nutrients [46].

Cadmium also induces depolarization of the root cell plasma membrane, thus reducing the driving force for cation uptake (e.g., potassium). Potassium plays a key role in determining the fate of cells. In both mammalian [47] and plant [48,49] systems, high cytosolic K^+^ concentrations are required to suppress the activity of caspase-like proteases and endonucleases and, hence, prevent programmed cell death (PCD). Therefore, it could be envisaged that when cadmium ions affect the absorption and efflux of potassium, the imbalance of potassium ions in the cytoplasm will be caused, thus activating the cell PCD process.

The toxicity threshold for Cd in plants varies between plant species. Cd concentration in grains has been found to increase proportionally to the total Cd content in soils, and 0.3 and 0.1 mg∙kg^−1^ levels are considered as a likely threshold for barley and wheat, respectively [14]. Necrosis symptoms can be observed in tomato when grown in half-strength Hoagland solution containing 10 μM Cd for two weeks [50]. At the same time, in some hyperaccumulator species concentrations of Cd in some tissues may exceed these values by several orders of magnitude (e.g., 15,000 mg∙kg^−1^ in the pith tissue in *Sedum alfredii*; [51]).

## 2. Current Trends in Breeding Programs

Given the importance of Cd toxicity issue for human health, the ideal solution for this problem would be via remediation of contaminated soils and preventing further anthropogenic-driven Cd contamination. However, achieving this goal may require a significant amount of time and orchestrated efforts and therefore can be considered as strategic only. An immediate solution may come through biological/genetic means, by creating cultivars with minimal, or no, Cd accumulation. Numerous attempts have been made to understand the genetic basis of Cd transport and accumulation in plants, in order to reduce Cd load. Given that over 50% of all calories consumed in the human diet comes from three major cereal crops (rice, wheat, and maize), reduction of Cd allocation to cereal grain is considered as the most essential objective in the breeding programs. This implies understanding the modes controlling Cd transfer from the roots to the shoots, followed by their modification by genetic means. Rice (*Oryza sativa* L.) is the main food feeding more than half of the global population; thus, the majority of research was done on this cereal. During the last decade, a series of QTL (quantitative trait loci) associated with Cd transportation in rice has been reported. Here, six different QTLs were found to be associated with Cd concentration in shoots during the seedling stage [52,53,54,55]. Genetic and physiological investigations suggest that the major QTL detected on the long arm of chromosome 11 is responsible for the specific translocation of Cd from the roots to the shoots [52]. Chromosome 7 harbors several putative metal transporters encoding genes such as *OsZIP8*, *OsHMA3*, and *OsNramp1* [42,56]. Three major QTL (*qCCBR1-1*, *qCCBR4-2*, and *qCCBR9-1*) associated with Cd concentration in brown rice and another one named *qCCMR11-2* in the milled rice explained more than 20% of the phenotypic variance respectively. These findings may potentially facilitate marker-assisted selection of rice varieties with low Cd accumulation in grain. It was shown that *OsCDT1*, *OsCDT3*, and *OsCDT4* located on chromosome 6, 1, and 2, respectively, encode a Cys-rich peptide in rice. This peptide chelates Cd at the cellular surface and prevents its further trans-membrane transportation, resulting in less cytosolic accumulation of Cd [57]. Further investigation demonstrated a differential binding affinity responding to diverse metal among CDTs [58], suggesting a possible genetic solution for modification of selectivity for these toxic metals. Recently, a new QTL, *qGCd7.1*, associated with high Cd accumulation was detected as a new *OsHMA3* allele [59]. However, this new allele was weak at both transcriptional and protein levels in the test genotypes compared with the fully functional *OsHMA3* lines. Liu et al. [60] also reported that a QTL for grain Cd concentration located on chromosome 7 (GCC7), which is responsible for differential shoot and grain Cd accumulation, had a different promoter activity of *OsHMA3* regulating the expression level in shoot and grain. Together, it is plausible that *GCC7* has a capacity to interact with *qGCd7.1* regulating the Cd uptake. Using genome-wide association studies, Liu et al. [61] reported 17 QTLs associated with grain Cd concentration. Among them, a novel candidate gene encoding *OsARM1*, a MYB transcription factor, previously known as responsible for freezing tolerance [62,63,64], was predicted to respond to Cd stress. Overexpression of the Cd-induced *MYB49* gene in *Arabidopsis* resulted in a significant increase in Cd accumulation [65]. MYB49 was also interacting with ABI5 which was up-regulated by Cd-induced ABA accumulation, implicating ABA signaling in control of Cd uptake and accumulation in plants [65]. In addition, one significant QTL referring to seed cadmium concentration was identified and validated in the F_6:7_ NILs in soybean [66]. This major QTL located on chromosome 9 was revealed to account for more than 50% of the genetic variation in RILs populations and contributed to a phenotype with low seed Cd concentration. Further, an effect of the *Cda1* locus on seed Cd concentration was confirmed by utilization of marker-selected soybean genotypes with a significant separation between high and low Cd accumulation [67]. However, despite these findings, no major progress in developing cultivars with low Cd-accumulating ability was achieved. The reasons for this are twofold. First, with so many QTLs reported, it is not practical to implement Cd tolerance traits without transferring some other undesirable genes. Second, at the physiological level, Cd transporters also operate as transporters of essential micronutrients such as Fe, Zn, or Mn. Thus, attempts to reduce Cd accumulations in plants may compromise their capacity for taking up these beneficial nutrients.

## 3. Molecular Mechanisms of Cd Uptake and Transport 

### 3.1. Cd Uptake by Roots

**Figure 2:**
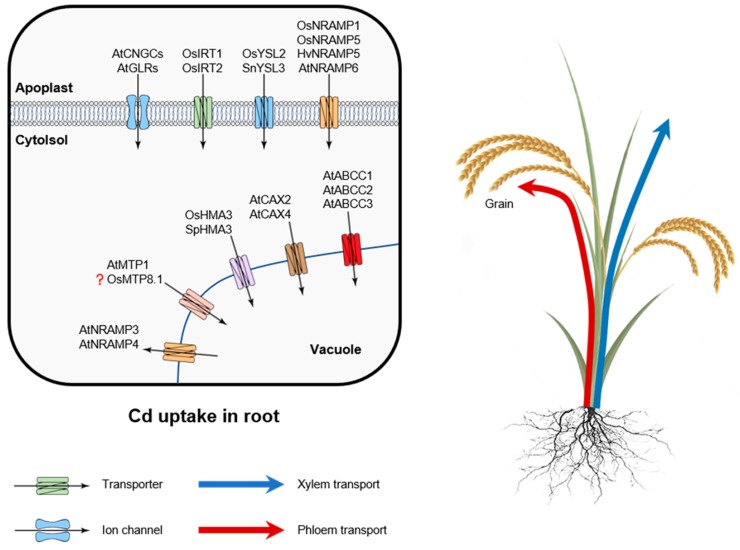
Uptake and intracellular compartmentation of cadmium in plant roots. Cd taken up by IRT1/2 (zinc/iron-regulated transporter-like protein), YSL2/3 (yellow stripe-like protein), and NRAMP1/5/6 (natural resistance-associated macrophage protein) transporters that are located at the plasma membrane of the root epidermis. Cd can be also transported into cells through non-selective cation (NSCC) channels such as CNGCs (cyclic nucleotide-gated channels) and GLRs (glutamate receptors). ABCC (ATP-binding cassette transporters), CAX (cation exchanger), HMA3 (metal-transporting ATPases), MTP1 (metal tolerance protein), and NRAMP3/4 mediate Cd transport and sequestration in the vacuole.

Plant cells have no Cd-selective transporter; hence, Cd uptake occurs through plasma membrane transporters involved in the uptake of other divalent cations, including Ca, Mg, Fe, Zn, Mn, and Cu [68]. Most of studies in the field were conducted on rice [69,70,71]. Several families of transporters have been implied to be responsible for Cd accumulation (Figure 2). 

When present in ionic form, Cd uptake by plant roots could be mediated by three major transport systems [40,72,73,74]. One of them is NRAMP (natural resistance-associated macrophage protein). The best-known family members are OsNRAMP1, OsNRAMP5, and AtNRAMP6. Cd can be also taken via ZIP (zinc/iron-regulated transporter-like protein) transporters, such as AtIRT1 and TcZNT1/TcZIP4, and finally, by low-affinity calcium transporters (such as TaLCT1 in wheat). Cadmium can be also transported by transport systems mediating plant Fe uptake. Two distinct strategies are used by plants to accumulate Fe from soils. The first one relies on the uptake of a reduced form of Fe(II) (so-called Strategy I). Strategy II is only observed in grasses and implies uptake of chelated Fe(III). Here, Fe(III)-phytosiderophore complexes (Fe(III)-PS) are taken into the root cells by yellow stripe 1 or yellow stripe-like 1 (YS1/YSL1) transporters identified in maize [75] and barley [76]. In chelated form, Cd may also be transported by YSL (yellow stripe-like 1) proteins such as OsYSL2 in rice and SnYSL3 in *Solanum nigrum* [41,77]. Plants belonging to Strategy I have the ability to transport Cd across plasma membrane by the same mechanisms employed for Fe^2+^ transport. OsIRT1 was shown to be implicated in Cd influx, for example, upon reaeration of soil after flooding [42,78]. In *Arabidopsis*, the rate of root Cd uptake was much lower in *atirt1* knockout mutant [79]. Ogawa et al. [78] showed that OsIRT1 and OsIRT2, two rice Fe^2+^ transporters, were also able to transport Cd when expressed in yeast. However, the role of these transporters for Cd uptake in planta seems to be rather minor in rice [73].

Of all the transporters above, NRAMP members have been demonstrated to be involved in numerous functions including uptake, translocation, intracellular transport, and detoxification of transition metals in many species [41,71,79,80,81,82,83]. In rice, OsNRAMP5, which plays a major role in Mn^2+^ uptake, may also represent a major route of Cd uptake by roots [73,82]. However, this view was challenged by Takahashi et al. [84] who showed rice mutants lacking functional OsNRAMP5 only showed a ~20% reduction in root Cd content, while accumulating significant amounts of Cd in the shoot. In barley, HvNRAMP5, which shares 84% identity with OsNRAMP5, can also mediate the uptake of Cd and Mn [83]. Interestingly, other orthologues of NRAMPs perform different specificity to NRAMP5. Among of them, OsNRAMP3 only has a capacity to transport Mn rather than Cd or Fe [85], while OsNRAMP1 can mediate uptake of trivalent Al ion [86]. In *Arabidopsis*, AtNRAMP3 and AtNRAMP4 can be functional to transport Cd^2+^ [87,88]. AtNRAMP6 is also involved in intracellular Cd^2+^ transport [40]. The above results indicate that plants can transport cadmium through these low affinity metal transporters. However, the genetic variation affecting the selectivity of the transport for specific metals needs to be further investigated. 

The summary of reported Cd transporters, their tissue-specific localization, and substrate specificity are given in the table below (Table 2).

In the roots of a halophytic plant *Suaeda salsa*, Cd^2+^ influx was inhibited by Ca^2+^ uptake blockers. Higher plants lack Ca^2+^-selective channels [90], and Ca^2+^ uptake in plants is believed to occur mainly via non-selective cation (NSCC) channels [89]. Thus, these results suggest that some Cd^2+^ can be transported into cells through NSCC. The molecular nature and mechanisms of regulation of Cd uptake via NSCC remain elusive. Altogether, *Arabidopsis* genome harbors 40 NSCC channels from two major groups; 20 of these channels are classified as “cyclic nucleotide-gated channels” (CNGC), and the other 20 as “glutamate receptors” [90]. The specific roles each of these members in Cd uptake require investigation.

### 3.2. Long-Distant Cd Transport

**Figure 3:**
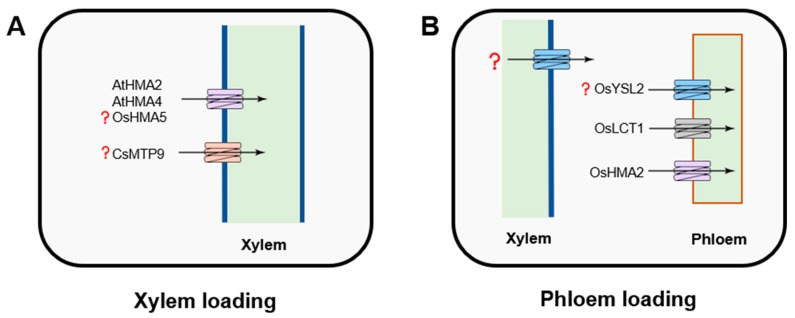
Transporters mediating xylem (**A**) and phloem (**B**) cadmium loading. HMA2 and LCT1 (low-affinity calcium transporter) are functioning in the translocation of Cd into the phloem. The major transporter that regulates xylem Cd loading is HMA2/4. The role of MTP9 in the long-distance Cd transport remains unclear.

Once taken by the root, Cd is then loaded into the xylem to be transported to the shoot. Cd translocation via the xylem is a key determinant of variation in grain Cd accumulation. The differences in the root-to-shoot Cd translocation rates of cultivars of *O. sativa* ssp*. indica* (high Cd-accumulating) and *O. sativa* ssp*. japonica* (low Cd-accumulating) rice explain the genotypic variation in Cd-accumulation observed in these two subspecies [26]. This translocation is mostly a function of retention in roots and xylem loading activity. The cation diffusion facilitator (CDF) family, otherwise known as the metal tolerance protein (MTP) family in plants, is believed to play an essential role in this process (Figure 3A). The MTPs/CDFs possess an ability to transport multiple ions and, depending on their selectivity, the members of MTP family are classified into three major clusters, including Zn-CDF, Mn-CDF, and Zn/Fe-CDF [97,98,99,100,117,118].

Functionally, MTP members operate as efflux transporters. It was shown that CsMTP9, known as an efflux transporter for Mn, rescued the Cd-hypersensitive phenotype when expressed in yeast, resulting from strengthened Cd^2+^ efflux activity [101]. However, OsMTP9 does not have a capacity to transport Cd, suggesting a different unidentified transporter involved in this pathway [102]. In rice, OsMTP8.1 has been described as a vacuolar Mn-specific transporter, but its role in Cd uptake has not been evaluated [103]. In *Arabidopsis*, MTP1 has been described as a Zn^2+^/H^+^ vacuolar transporter, which also showed a capacity to mediate Cd flux in *Thlaspi goesingense* [99,104]. Podar et al. [119] also reported that MTP1 is a Zn^2+^/Co^2+^-specific transporter, with a high affinity to Zn^2+^ when expressed in barley. Importantly, the structure–function analysis of MTP1 suggests that it is possible to increase the selectivity of AtMTP1 towards Zn by modifying a five-residue sequence within the MTP1 N-segment of the His-rich intracytoplasmic loop. This His-rich loop was suggested to be a determinant of the identity of the metal ion [100]. 

P_1B_-type heavy metal-transporting ATPases (HMAs) transport heavy metals (Cu^+^, Cu^2+^, Zn^2+^, Co^2+^, Cd^2+^, Pb^2+^) across membranes are present in most organisms and crucial for the cellular metal homeostasis. Most of them are not fully characterized. OsHMA5 has also been localized to the root pericycle cells and xylem but seems to have more affinity to copper rather than other metal ions [105]. In *Arabidopsis*, HMA2 and HMA4 are considered to load Cd and Zn into the xylem [106,107]. The latest study on the identification of mutations from amino acids of HMA4 transmembrane helices (TMs) indicated a potential solution to alter the function of this transporter in *Arabidopsis hma2/hma4* double mutant. The transporter with substitution of E169 and K667 by Ala residues can still transport Cd^2+^ as the native protein, while Zn^2+^ transport is completely silenced [107]. 

Although the long-distant transport via phloem is crucial for Cd accumulation in seeds and grains, the mechanistic basis of this process is poorly understood (Figure 3B). So far, OsHMA2 has been described to function in intervascular Cd delivery to developing tissues in the uppermost node of rice [85]. The loss of OsHMA2 function in insertion mutants results in decreased leaf and grain Cd concentrations [120]. An increase in Cd tolerance in rice and reduction of Cd accumulation in grains can be observed in the overexpression lines of OsHMA3 [54,121]. Another transporter that mediates phloem-based Cd distribution is LCT1 (low-affinity cation transporter 1) [74,108]. *OsLCT1* encodes a plasma membrane-localized protein, which is mainly expressed in phloem parenchyma cells in leaf blades and nodes during the reproductive stage [108]. *OsLCT1* transcript level is strongly upregulated during the reproductive stage in rice, and a decrease in Cd concentration in grains can be observed in its *oslct1* knockdown line [74]. *OsYSL2*, an orthologue of *ZmYSL1*, is mainly expressed in parenchyma, suggesting a potential capacity to transport Cd in phloem [77]. At this stage, given the limited knowledge on molecular mechanisms of phloem loading, *LCT1* and *HMA2* are the likely candidate genes for further modification to restrain the translocation of Cd, particularly into grain in cereal crops.

## 4. Tissue Tolerance Mechanisms

To minimize the toxicity of Cd exposure and its accumulation, plants have evolved various detoxification mechanisms to surmount the adverse effects. Sequestration in root vacuoles has been demonstrated as the major process limiting the translocation of Cd to shoots and seeds [54,122]. As the largest organelle inside the mature plant cell, vacuole is an important reservoir of ions and metabolites and is crucial for the detoxification process as well as normal cell development [123,124].

Cd has a strong affinity for thiol-containing molecules such as cysteine, glutathione, and phytochelatins (PCs). Phytochelatins react with heavy metal ions by glutathione S-transferase catalyzation in the cytosol, and afterwards, they are sequestered into the vacuole for degradation. In sequestration into vacuoles, ATP-binding cassette transporters mediate influx of PC-metal (loid) complexes. In *Schizosaccharomyces pombe*, the first ABC transporter HMT1 located at the tonoplast was reported to aid transporting PC-Cd complexes formed in the cytosol [125,126,127]. A functional homolog of HMT1 has been then reported in *Caenorhabditis elegans* and *Drosophila* [128,129]. In *Arabidopsis*, ABCC1 and ABCC2 have been identified as the main transporters mediating PC uptake into vacuoles [109,130]. Cd staining experiments showed that Cd was mainly present in the cytosol in *abcc1abcc2* double mutant, whereas almost all Cd has been compartmentalized in the vacuoles of the wild type [131]. Orthologs of AtABCC1 have been identified in numerous grasses including rice, maize, and barley. In barley, a vacuole kinetic analysis also suggests that this transport mechanism is conserved across species [132]. AtABCC3 was also shown to play an important role on Cd tolerance. Plants overexpressing *AtABCC3* showed Cd-tolerant phenotypes, while a mutant defective in *atabcc3* was more sensitive to Cd stress. The function of AtABCC3 is highly dependent on PCs. When AtABCC3 was expressed in the *cad1-3* mutant (defective in PC synthesis) or AtABCC3-overexpressing plants were subjected to BSO (an PC biosynthesis inhibitor), the Cd-tolerant phenotype disappeared [110].

In contrast to ABCCs, for which Cd transport is coupled to phytochelatins, the heavy metal-transporting ATPases (HMAs), cation diffusion facilitator (CDF), and Ca^2+^ exchangers transport the ionic form of Cd (Cd^2+^). P1B-type ATPases have also been shown to play a key role on sequestering Cd in root vacuoles [54]. Of interest is the fact that OsHMA3 seems to be highly specific for Cd, while the *Arabidopsis halleri* HMA3 has a preference for Zn. However, the *A. thaliana* HMA3 shows broad substrate specificity being able to transport Co, Pb, Cd, and Zn [111,112]. OsHMA3, a vacuolar P1B-type ATPase was found to contribute 85.6% of the variance in Cd content between low-and high-cadmium accumulation varieties of rice [54]. The important role of OsHMA3 as controlling shoot Cd accumulation was demonstrated by QTL analysis of low and high Cd-accumulating rice cultivars [133]. In *Arabidopsis*, the function of HMA3 was attributed to limiting long-distance transport of Cd from the root to the shoot as this transporter is predominantly expressed in the root and thus operates in its sequestration here [113]. The crucial role of HMA3 for plants’ ability to handle high concentrations of Cd in shoots is also broadly reported for hyperaccumulating species. Working with hyperaccumulating *Sedum alfredii* species, Zhang et al. [134] reported high level of *SaHMA3h* expression leading to efficient detoxifying ability in this species. *S. alfredii* plants also had a larger number of gene copies as compared with its non-hyperaccumulator ecotype. In *S. plumbizincicola* shoots, particularly in young leaf cells, SpHMA3 is critical for Cd detoxification and acts by sequestering Cd into vacuoles [114]. Some transporters like CAXs and NRAMPs, which are tonoplast-localized, also play a role in Cd tolerance [135]. Most of the CAXs are Ca^2+^ specific, but in *Arabidopsis*, AtCAX2 and AtCAX4 were shown to have the ability to transport other metals like Cd, Zn, and Mn [115,116]. A heterologous overexpression of SaCAX2a from hyperaccumulating *S. alfredii* plants in tobacco resulted in improved Cd tolerance [134]. Likewise, the *atnramp3atnramp4* double knockout mutant exhibits increased sensitivity to Cd [136] but also displayed Fe deficiency symptoms. NRAMP3 and NRAMP4 are also believed to be involved in the remobilization of essential metals by exporting them across the tonoplast membrane from the vacuole [137]. 

Besides those divalent cation transporters functioning in cellular sequestration, organic acids are considered to have an involvement in detoxification mechanisms. Of interest is that organic acids such as citric, malic, and carboxylic acids may stabilize Cd^2+^ in a complex form. The root hair of Cd hyperaccumulator *Thlaspi caerulescens* contains high levels of citric and malonic acids that distribute high Cd levels in cell walls, ensuring that plants can tolerate Cd-induced stress [138]. In leaves of the Cd hyperaccumulator *Solanum nigrum*, both acetic and citric acid bind with Cd, enabling the formation of Cd-organic acid complex for chelation [139]. Ehsan et al. [140] found that citric acid in culture solution forms a citric complex of Cd that can be easily translocated from the roots to shoots of *Brassica napus* without any toxicity occurrence, thus peaking its phytoextraction potential. The secretion of malic and oxalic acids in tissues of *Agrogyron elongatum* grown in the nutrient medium was positively correlated with peak Cd levels and tolerance mechanisms for coping with Cd toxicity [141]. In addition, the binding affinity of organic acids to Cd forms a complex with Cd^2+^ thus reducing the availability of free toxic Cd^2+^ in the growth medium and alleviates phytotoxicity induced by excess Cd.

## 5. Conclusions and the Way Forward

Cd has long been recognized as a major health threat to humans. It represents one of the most toxic substances released into the environment, and practically all human populations are environmentally exposed to Cd, mostly through plant-derived food. Thus, the ability to regulate Cd loading into the xylem and its delivery to the shoot with a transpiration stream in leafy vegetables is critical to avoid Cd entering the food chain. Equally important is the prevention of Cd being accumulated in cereal grains (by phloem-based retranslocation mechanism). While a significant progress has been made in revealing the molecular nature of transporters mediating Cd uptake and transport across cellular membranes, reducing Cd accumulation in plants is complicated by the fact that most known Cd transporters also operate in the transport of essential nutrients such as Zn, Fe, Mn, or Cu. Thus, the real progress in the field may be only achieved if transport of these nutrients and Cd is uncoupled. This calls for more studies dealing with structure–function relations of various transporters isoforms. In this context, Kawachi et al. [98,100] conducted a structure–function analysis of *Arabidopsis* MTP1 transporter from the cation diffusion facilitator family. Based on the crystal structure of the *Escherichia coli* and using site-directed mutagenesis, they showed that Ala substitution of either Asn258 in TM5 or Ser101 in TM2 reduced AtMTP1 selectivity for Zn^2+^ in yeast. They also showed that deletions in N-terminal and His-rich intra-molecular cytosolic domains and mutations of single residues flanking the transmembrane pore or participating in intra- or inter-molecular domain interactions selectively affected yeast’s ability to accumulate Co and Cd [100]. Thus, uncoupling of Cd transport from transport of essential micronutrients is technically possible and just required some more orchestrated efforts. The major hurdle in this process is a high tissue specificity of transporter operation. Two tissues are of a specific importance in this context. The first one is a xylem parenchyma in plant roots, a major “controller” of Cd loading into the xylem and its transport to the shoot. The second one is a phloem tissue that operates in the last step of the metal transport from mature leaves (where Cd is delivered) to the developing grains. Ion remobilization from leaves and transport to developing grains is currently terra incognita, hinting at many unexploited ways to improve crops genetically. Another promising and currently underexplored avenue is to understand the role of non-selective cation channels in Cd uptake and reveal mechanisms of their regulation. *Arabidopsis* genome harbors 40 non-selective cation channels (NSCC) [90]; 20 of them belong to ionotropic “glutamate receptor-like” (GLR) family. Other plant families also have over 10 GLR members in their genomes [93,94,95,96]. GLRs are believed to be tetramers consisting of different subunits, with preferential expression in root tissues [142]. The other 20 channels are classified as “cyclic nucleotide-gated channels” (CNGC). CNGCs are structurally similar to members of the superfamily of six transmembrane “Shaker-like” pore-loop ion channels [91,92]. All these channels need to be functionally characterized for their ability to uptake Cd^2+^ in functional (MIFE or patch-clamp electrophysiology) assays and then validated in genetic studies. 

## Figures and Tables

**Table 1:** Cd content in leafy vegetable plants (selected examples).

Leafy Vegetable Species	Range of Cd in Shoot (mg∙kg^−1^)	Mean or Range of Cd in Soil (mg∙kg^−1^)	Reference
*Lactuca sativa* L.	5.8 to 9.1	0.12 to 0.31	Baldantoni et al. [32]
*Cichorium endivia* L.	0.61 to 3.80	0.13 to 0.51	Baldantoni et al. [32]
*Brassica pekinensis* L.	1.05 to 3.51	2.42	Wang et al. [33]

**Table 2:** Transporters mediating cadmium uptake and translocation in plants.

*Transporter*	*Localization*	*Function/Substrate*	*Reference*
OsNRAMP1	Roots and shoots (PM)	Influx of Cd, Al	[42]
OsZIP8	Root (PM)	Influx of Zn, Fe	[52]
OsHMA3	Roots (tonoplast)	Cd sequestration in root vacuoles	[52]
SnYSL3	Vascular tissues and epidermal cells of the roots and stems (PM)	Transport of nicotianamine complexes containing Fe(II), Cu, Zn, and Cd	[41]
OsYSL2	Vascular bundles, Roots (PM)	Influx of nicotianamine complexes containing Fe(II), Mn, Ni, and Cd	[77]
OsIRT1	Roots (PM)	Uptake of Fe, Zn, Mn, and Cd	[78]
OsIRT2	Roots (PM)	Uptake of Fe, Zn, Mn, and Cd	[78]
OsNRAMP5	Roots (PM)	Uptake of Mn and Cd	[82]
HvNRAMP5	Roots (PM)	Uptake of Mn and Cd	[83]
OsNRAMP3	Vascular bundles, roots, leaves (tonoplast)	Uptake of Mn	[85]
AtNRAMP3	Vascular bundles, roots, leaves (tonoplast)	Efflux of Fe and Cd	[87,88]
AtNRAMP4	Vascular bundles, roots, leaves (tonoplast)	Efflux of Fe and Cd	[87,88]
AtNRAMP6	Roots, young leaves (PM)	Influx of Mn,	[40]
AtCNGCs	Roots (PM)	Transporter for multiple cations	[89,90,91,92]
AtGLRs	Roots (PM)	Transporter for multiple cations	[89,90,93,94,95,96]
AtMTP1	Roots and leaves (tonoplast)	Transporter for Zn and Cd	[97,98,99,100]
CsMTP9	Roots endodermal cells (PM)	Efflux of Mn and Cd	[101]
OsMTP9	Roots (PM)	Efflux of Mn	[102]
OsMTP8.1	Roots (tonoplast)	Sequestration of Mn into vacuoles	[103]
TgMTP1	Roots and leaves (tonoplast)	Transporter for Zn and Cd	[104]
OsHMA5	Roots, vascular bundles (tonoplast)	Loading of Cu in xylem	[105]
AtHMA2	Roots, vascular tissue (PM)	Delivery of Zn and Cd to xylem	[106,107]
AtHMA4	Roots, vascular tissue (PM)	Delivery of Zn and Cd to xylem	[106,107]
OsLCT1	Leaves, nodes, phloem parenchyma (PM)	Efflux of Cd, Ca, Mg, and Mn	[74,108]
AtABCC1	Roots and shoots (tonoplast)	Uptake of PCs	[109]
AtABCC2	Roots and shoots (tonoplast)	Uptake of PCs	[109]
AtABCC3	Roots and shoots (tonoplast)	Uptake of PCs	[110]
AhHMA3	Roots, shoots (tonoplast)	Sequestration of Zn into vacuoles	[111]
AtHMA3	Vascular tissues (tonoplast)	Transport of Zn, Co, Pb, and Cd	[112,113]
SpHMA3	Roots, shoots (tonoplast)	Sequestration of Cd into vacuoles	[114]
AtCAX2	Roots (tonoplast)	Vacuolar Cd, Zn, and Mn transport	[115,116]
AtCAX4	Roots (tonoplast)	Vacuolar Cd, Zn, and Mn transport	[115,116]

*Abbreviations*: PM, plasma membrane.
